# Bamboo Leaf Flavonoids Suppress Oxidative Stress-Induced Senescence of HaCaT Cells and UVB-Induced Photoaging of Mice through p38 MAPK and Autophagy Signaling

**DOI:** 10.3390/nu14040793

**Published:** 2022-02-14

**Authors:** Yanpei Gu, Fan Xue, Hongrui Xiao, Lihuan Chen, Ying Zhang

**Affiliations:** Zhejiang Key Laboratory for Agro-Food Processing, Zhejiang Engineering Center for Food Technology and Equipment, College of Biosystems Engineering and Food Science, Zhejiang University, Hangzhou 310058, China; yanpeigu@zju.edu.cn (Y.G.); xf21713038@zju.edu.cn (F.X.); xhrfys@163.com (H.X.); 18861879882@163.com (L.C.)

**Keywords:** skin aging, bamboo leaf flavonoids, cellular senescence, p38 MAPK, autophagy

## Abstract

With the global escalation of the aging process, the research on aging mechanisms and anti-aging strategies has become a hot spot. As the most external organ of the human body, skin can be used as an ideal organ for the study of endogenous and exogenous aging. Bamboo leaf flavonoids (BLF) possess a variety of biological effects such as antioxidant, anti-bacterial, anti-inflammatory, lipid-lowering, anti-radiation, and anti-aging. However, it is still unclear whether they can delay skin aging. This study aimed to analyze the inhibitory effect of BLF on skin aging and explore their molecular mechanisms. We found that 10–40 μg/mL BLF significantly inhibited the senescence of HaCaT cells induced by AAPH, which might be related to their antioxidant and anti-inflammatory abilities. Further mechanism studies showed that mitogen-activated protein kinase (MAPK), especially the p38 MAPK pathway, was the key to BLF to alleviate the senescence of HaCaT cells. In addition, autophagy was also involved in the anti-senescence effect of BLF. The results were also verified in UVB-induced photoaging mice. Therefore, BLF can be used as a potential therapeutic agent to intervene skin aging in vitro and in vivo.

## 1. Introduction

Aging is a comprehensive manifestation of decreased functions and physical disorders during the degenerative period, and it will increase the risk of age-related diseases (ARDs) such as neurodegenerative diseases, cardiovascular diseases, diabetes, osteoarthritis, and cancers. Research on the mechanism of aging and how to prevent and delay the occurrence and development of ARDs will help improve the quality of life of people and reduce the economic burden of society and families, which has gradually become a hotspot for scientists. The aging of the skin is the most intuitive manifestation of the aging of the body, such as increased wrinkles, relaxation, roughness, and abnormal pigmentation [1]. Unlike other organs of the human body, the aging of skin is not only affected by genetic factors but also by environmental factors. UV radiation and long-term exposure to cigarette smoke are independent causes of accelerated skin aging [2,3]. Therefore, skin can be used as an ideal organ for studying aging, whether it is endogenous aging or exogenous aging [1].

The free radical theory of aging is widely considered to be one of the core mechanisms mediating skin aging [4]. Oxidative stress caused by the accumulation of reactive oxygen species (ROS) generated by mitochondria or ultraviolet radiation can directly destroy lipids, proteins, and nucleic acids, inter alia via protein carbonylation and the production of 8-hydroxy-2′-deoxyguanosine (8-OHdG) [5,6]. In addition, excessive ROS can regulate the mitogen-activated protein kinase (MAPK) and nuclear factor kappa-B (NF-κB) signaling pathway, leading to the activation of heterodimer activator protein 1 (AP-1) and NF-κB. Activated AP-1 can induce the production of matrix protein metalloenzymes (MMPs) to degrade collagen and elastin in skin tissues [7]. Activated NF-κB can promote the expression of proinflammatory cytokines such as IL-1β, COX-2, and TNF-α to regulate the inflammatory response and further accelerate the occurrence of skin aging [8]. At present, there have been many studies on the use of oxidants such as H_2_O_2_ and AAPH to induce cellular senescence [9,10].

Autophagy is an evolutionarily conserved degradation mechanism that exists widely in yeasts to mammals [11]. More and more evidence suggests that autophagy is closely related to aging and ARDs. At the same time, autophagy is closely related to skin aging [1,12]. DNA damage and senescence of autophagy-deficient keratinocytes are increased abnormally after oxidative stress [13]. Inhibition of autophagy can also lead to the premature senescence of fibroblasts [14]. On the contrary, the activation of autophagy inhibits stress-induced cellular senescence and has the effect of delaying aging. Caffeine can protect the skin from oxidative stress-induced senescence by regulating the A2AR/SIRT3/AMPK pathway to activate autophagy [10]. Astragaloside exerts anti-photoaging effects in UVB-induced premature senescence of rat dermal fibroblasts through enhanced autophagy [15]. In addition, the MAPK pathway is involved in the regulation of autophagy. The induction of autophagy by resveratrol is related to p38 MAPK signaling [16]. Doxycycline ameliorates autophagy by inhibiting p38 MAPK in cardiac myocytes [17].

Flavonoids are a class of secondary metabolites produced by plants in order to resist stress behaviors such as pathogen invasion, ultraviolet radiation, and physical damage, which have many benefits. The main active components of bamboo leaf flavonoids (BLF) isolated from bamboo leaves are four carbon glycoside flavonoids, namely orientin, isoorientin, vitexin, and isovitexin (Figure S1) [18]. Studies have shown that BLF have a variety of biological effects such as antioxidant, lipid-lowering, anti-radiation, anti-bacterial, anti-inflammatory, anti-aging, etc., and have been approved by the National Health and Planning Commission of China as a “new food ingredient” [19,20]. However, the role of BLF in reducing or inhibiting cellular senescence and photoaging has been rarely reported. HaCaT cells are isolated from the spontaneous transformation of human keratinocytes and are very close to normal keratinocytes and easy to be cultured. It is widely believed that HaCaT cells maintain complete epidermal differentiation and non-tumorigenicity, and they are a suitable and stable cell model for the study of skin [21]. In this study, we treated HaCaT cells with 1 mM AAPH to induce senescence according to our previous research, and on this basis, we analyzed the anti-aging effect and mechanism of BLF. In addition, the results were verified on a mouse model of UVB-induced dorsal skin photoaging.

## 2. Materials and Methods

### 2.1. Chemicals and Antibodies

BLF (the mass fraction of orientin, isoorientin, vitexin, and isovitexin was 80.66%, Figure S2) were prepared in the laboratory. AAPH, rapamycin (Rapa), and 3-methyladenine (3-MA) were obtained from Sigma (St.Louis, MO, USA). Dehydrocorydaline (DE) and SB203580 (SB) were obtained from MedChemExpress (Deer Park, NJ, USA). Reagents required for cell culture were purchased from HyClone (Logan, UT, USA) and Gibco (Grand Island, NY, USA), respectively. Anti-JNK1/2/3 and p-JNK1/2/3 (Thr183/Thr183/Thr221) antibodies were purchased from Beyotime biotechnology (Shanghai, China); anti-K9M-H3, IL-10, and Alexa fluor 488-conjugated goat anti-rabbit IgG (H+L) antibodies were purchased from ABclonal technology (Wuhan, China); anti-p16, p21, COX-2, LC3A/B, Beclin-1, SQSTM1/p62, p38, p-p38 (Thr180/Tyr182), ERK1/2, p-ERK1/2 (Thr202/Tyr204), ULK1, MMP-3, COL1A1, GAPDH, and anti-rabbit IgG, HRP-linked antibodies were obtained from Cell signaling technology (Beverly, MA, USA).

### 2.2. Cell Culture

HaCaT cells were obtained from the Cell Bank of the Chinese Academy of Sciences. The resuscitated HaCaT cells were cultured in DMEM high-glucose medium containing 15% fetal bovine serum and 1% antibiotics (penicillin, streptomycin) and placed in a humidified incubator at 37 °C and 5% CO_2_. Approximately, 80% confluent HaCaT cells were digested with a 0.25% Trypsin/0.02% EDTA solution and passaged, and the logarithmic growth phase cells were taken for experiments.

### 2.3. Animals, UVB Irradiation, and Treatments

Forty 2-month-old female Kunming mice were purchased from The Animal Experimental Center of Zhejiang Chinese Medicine University (Hangzhou, China). All mice were kept at room temperature (24 ± 1 °C) and under a 12 h light/dark cycle and were freely provided with water and standard forage (5% fat, 45% carbohydrate, and 21% protein). After one week of acclimation, mice were randomly divided into 4 groups: control, UVB alone, UVB + 2.5% BLF, and UVB + 5.0% BLF (the specific formula is shown in Table S1). Mouse dorsal skin was shaved and irradiated with a UVB irradiator (Sigma, Shanghai, China) for 8 weeks. The dose of UVB was equivalent to 60 mJ/cm^2^ in the first two weeks, increased to 120 mJ/cm^2^ in the third week, 180 mJ/cm^2^ in the fourth week, and 240 mJ/cm^2^ in the 5~8 weeks. The total irradiated dose was approximately 6.9 J/cm^2^.

During UVB irradiation, 2.5% and 5.0% BLF were applied to mice 5 times a week. Then, 24 h after the last irradiation, the mice were anesthetized and killed by cervical dislocation. Skin tissue in the depilated area of the back was collected, connective tissue and subcutaneous fat were stripped and then fixed with 4% paraformaldehyde solution and 2.5% glutaraldehyde solution or rapidly frozen in liquid nitrogen and stored at −80 °C until further use.

### 2.4. Assessment of Cell Proliferation

HaCaT cells were seeded into the 96-well cell culture plates at a density of 5 × 10^3^ cells/well. Different doses of BLF (5–160 μg/mL) and 1 mM AAPH were added after the cells were adherent overnight. At the same time, a blank group containing only the medium and a negative control group containing only cells without AAPH and BLF were set. After incubating for 48 h, it absorbed the supernatant, and we added the fresh medium containing 10 μL 3-(4,5-dimethylthiazol-2-yl)-2,5-diphenyltetrazolium bromide (MTT) solution (Beyotime, Shanghai, China) to each well. After incubation in the incubator for 4 h, the culture medium was absorbed and shaken with DMSO solution for 10 min. The absorbance of each well was measured at a wavelength of 490 nm using a microplate reader (BioTek, Winusky, VT, USA). The cell proliferation inhibiton rate (%) = (OD negative control − OD treatment)/(OD negative control − OD blank) × 100.

### 2.5. Determination of ROS Level

HaCaT cells were seeded into the 6-well cell culture plates at a density of 1 × 10^5^ cells/well. After treatment with different doses of BLF (10, 20, and 40 μg/mL) and 1 mM AAPH for 48 h, HaCaT cells were harvested and incubated in 10 µM DCFH-DA (Beyotime, Shanghai, China) solution diluted in serum-free medium for 20 min at 37 °C. Then, the cells were washed three times with serum-free medium to sufficiently remove DCFH-DA that did not enter the cells. The level of ROS was measured using a flow cytometer under the FITC channel (exciter 488 nm, emitter 525 nm).

### 2.6. Analysis of Antioxidant Enzymes and Oxidative Damage Markers

The activities of superoxide dismutase, catalase, and glutathione peroxidase were respectively determined by a total SOD assay kit with WST-8, catalase assay kit, and cellular glutathione peroxidase assay kit with NADPH (Beyotime, Shanghai, China). Lipid oxidative damage marker malondialdehyde was detected using a lipid oxidation (MDA) assay kit (Beyotime, Shanghai, China). Protein carbonylation, DNA oxidative damage marker 8-OHdG, and IL-2 were detected using a protein carbonyl content detection kit (Solarbio, Beijing, China), human 8-OHdG ELISA kit, and human IL-2 ELISA kit (Mlbio, Shanghai, China). HaCaT cells were seeded into the 6-well cell culture plates at a density of 1 × 10^5^ cells/well. After treatment with different doses of BLF (10, 20, and 40 μg/mL) and 1 mM AAPH for 48 h, HaCaT cells were harvested and lysed; then, they were centrifuged at 12,000× *g* for 10 min at 4 °C. Take the supernatant for determination. All steps were performed according to the manufacturer’s instructions.

### 2.7. Confocal Fluorescence Microscopy

The formation of SAHF in cultured cells was detected by immunofluorescence. Briefly, after treatment, HaCaT cells were fixed and permeabilized at room temperature with 4% paraformaldehyde and 0.5% Triton-X-100 solution, respectively. After washing with PBS three times, cells were blocked by PBS containing 3% goat serum and 1% BSA for 1 h, which was followed by adding the prepared primary antibody specific for K9M-H3 and incubating at 4 °C overnight. Then, cells were incubated with secondary antibody specific for Alexa Fluor 488-conjugated Goat Anti-Rabbit IgG (H+L), and nuclei were marked by 1 µg/mL 4’,6-diamidino-2-phenylindole (DAPI) (Sigma, St.Louis, MO, USA) solution at the same time. Cells with condensed K9M-H3 that co-localized with DAPI in the nuclei were considered as SAHF positive, and the percentage of SAHF positive cells relative to the total number of cells was calculated. For detecting mitochondrial membrane potential, HaCaT cells were incubated with JC-1 staining solution (Beyotime, Shanghai, China) prepared according to the instructions at 37 °C for 20 min and then washed twice with JC-1 staining buffer. After the above treatments and steps, photographs were captured using the confocal microscope (Leica, Wetzlar, Germany).

### 2.8. mRNA Sequencing and Quantitative Real-Time PCR (qRT-PCR)

The total RNA of each group of cells was extracted by Trizol reagent (Invitrogen, Carlsbad, CA, USA). After detecting the concentration, purity, and integrity of RNA, mRNA sequencing based on an Illumina Novaseq 6000 platform was carried out at Majorbio Co. Ltd. (Shanghai, China). The sequenced reads were aligned against the Homo sapiens genome by using the Human GRCh38 transcript set. The transcript-level counts were imported and analyzed for differential expression by using the software DESeq2. For qRT-PCR, the reverse transcription kit (Takara Bio Inc., Shiga, Japan) was used for reverse transcription. The mixtures containing PowerUp SYBR Master Mix (Applied Biosystems, Carlsbad, CA, USA), cDNA, and primers (Sangon Biotech, Shanghai, China) at optimal concentration were amplified using a fluorescence quantitative PCR instrument (Life Technologies, Carlsbad, CA, USA). GAPDH was used as the reference gene, and the 2^−ΔΔCt^ method was used to calculate the relative mRNA expression of each group of cells. The primer sequences were: F-5’-CGGAGTCAACGGATTTGGTCGTAT-3’, R-5’-AGCCTTCTCCATGGTGGTGAAGAC-3’ for GAPDH; F-5’-TAGCAGCGGAACAAGGAG-3’, R-5’-AAACGGGAACCAGGACAC-3’ for p21; F-5’-ACCAGAGGCAGTAACCATGC-3’, R-5’-CCTGTAGGACCTTCGGTGAC-3’ for p16; F-5’-TAGTGTGGTGGTGCCCTATG-3’, R-5’-CCAGTGTGATGATGGTGAGG-3’ for p53; F-5’-AAAAGACAACTCTCGTCGCAT-3’, R-5’-CCGCTTTCCTCTAGTTGTACG-3’ for Lamin B1.

### 2.9. siRNA Transfection

HaCaT cells were seeded into the 6-well cell culture plates at a density of 1 × 10^5^ cells/well for 24 h to reach around 70% confluence before transfection. Lipofectamine^TM^ 3000 reagent (Life Technologies, Carlsbad, CA, USA) and siRNA were diluted in serum-free medium respectively, incubated at room temperature for 5 min, and then mixed and incubated at room temperature for 20 min to prepare siRNA-liposome mixture and then added drop by drop into cells for 6 h. Medium containing the transfection mixture was replaced with a complete medium containing serum for another 18 h incubation before AAPH and BLF treatment followed by various analyses. siRNA sequences used here were siULK1#1 F-5’-GGUACCUCCAGAGCAACAUTT-3’, R-5’-AUGUUGCUCUGGAGGUACCTT-3’; siULK1#2 F-5’-CCUGUGACACAGACGACUUTT-3’, R-5’-AAGUCGUCUGUGUCACAGGTT-3’; siULK1#3 F-5’-CCAAGUGCAAGCUGUGCAUTT-3’, R-5’-AUGCACAGCUUGCACUUGGTT-3’ (Tsingke Biotechnology, Beijing, China).

### 2.10. Western Blotting

Briefly, after treatment, RIPA Lysis buffer supplemented with protease inhibitors (Beyotime, Shanghai, China) was used to extract the total protein of each group of skin tissues and HaCaT cells. The protein concentration was quantified using the BCA protein assay kit (Beyotime, Shanghai, China). After separation by polyacrylamide gel electrophoresis, the protein was transferred to PVDF membrane. Blots were incubated with different antibodies (K9M-H3, p21, p16, JNK1/2/3, p-JNK1/2/3 (Thr183/Thr183/Thr221), p38, p-p38 (Thr180/Tyr182), ERK1/2, p-ERK1/2 (Thr202/Tyr204), IL-10, COX-2, LC3A/B, Beclin-1, SQSTM1/p62), washed, and incubated with a horseradish peroxidase (HRP)-labeled secondary antibody. We visualized the bands by using an ECL commercial kit (Beyotime, Shanghai, China) for chemiluminescence. Taking GAPDH as the reference protein, Image J software was used to analyze the gray value to quantify protein expression.

### 2.11. Measurement of Skin Hydration

The skin hydration was measured by the direct drying method [22,23]. Briefly, about 0.2 g of skin was quickly cut, weighed accurately and quickly put into the oven to dry to constant weight at 105 °C. Skin hydration percentage (%) = (wet weight − dry weight)/wet weight × 100.

### 2.12. Histopathological Analysis

After being fixed with paraformaldehyde solution, the skin tissue was dehydrated by gradient ethanol, embedded in paraffin, and sectioned. Hematoxylin and eosin staining was performed to observe changes in epidermal thickness. Masson staining was used to detect changes in collagen fibers, which were stained blue [24,25]. Immunohistochemical staining was performed to analyze the expression of MMP-3 and COL1A1 protein. Histological observations were performed using a binocular optical microscope (Nikon, Tokyo, Japan).

### 2.13. Transmission Electron Microscopy (TEM)

Mouse dorsal skin tissue and HaCaT cells were fixed with 2.5% glutaraldehyde solution overnight at 4 °C. After fixation, skin tissues or cells were treated with 1% osmic acid for 2 h, washed with PBS, and dehydrated with increasing concentrations of ethanol and pure acetone. Following embedding by Spurr, the cells were sliced into ultrathin sections. Then, after staining with lead citrate and uranyl acetate solutions for 10 min, respectively, photographs were taken using Hitachi H-7650 transmission electron microscopy (Hitachi, Tokyo, Japan).

### 2.14. Data Analysis and Statistics

All experiments were repeated at least three times, and the data were expressed as mean ± standard deviation (SD). Statistical analyses were performed by SPSS 19.0, significant differences between the groups were determined by using a one-way analysis of variance (ANOVA), taking *p* < 0.05 as significant difference and *p* < 0.01 as extremely significant difference.

## 3. Results

### 3.1. BLF Inhibits Oxidative Stress-Induced Senescence

**Figure 1:**
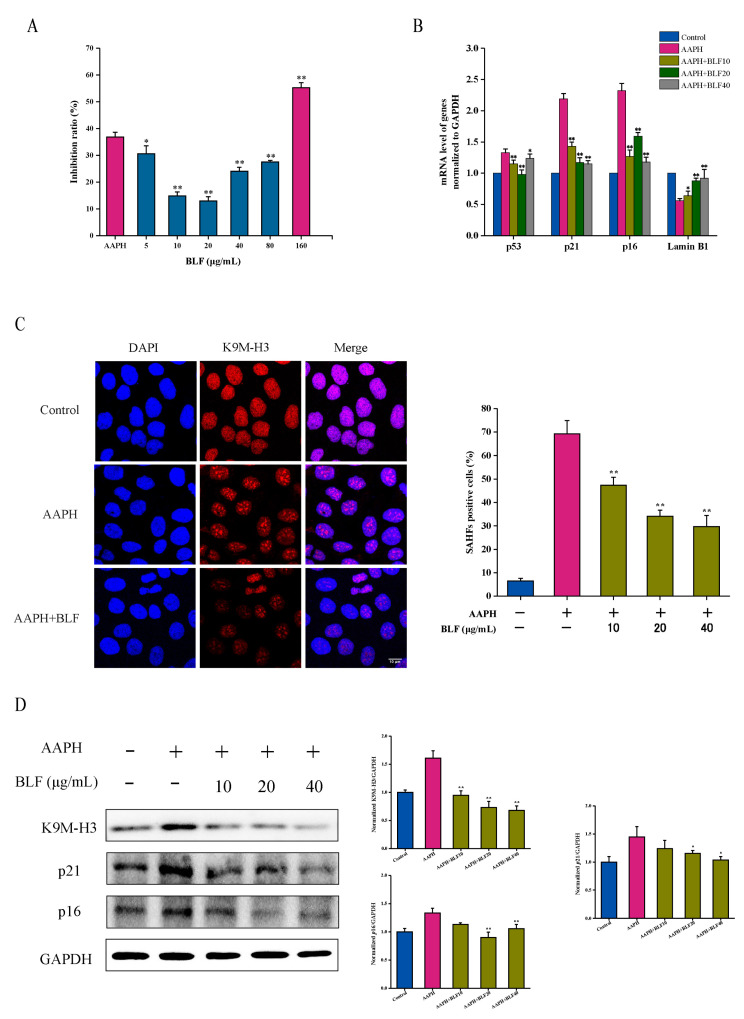
BLF attenuated AAPH-induced senescence of HaCaT cells. (**A**) Effects of different doses of BLF and 1 mM AAPH treatment for 48 h on the proliferation inhibition of HaCaT cells determined by the MTT assay. (**B**) Gene expression of p53, p21, p16, and Lamin B1 was determined by qRT-PCR. (**C**) Representative images of SAHF formation photographed by confocal microscope. Scale bar = 10 μm. The percentage of SAHF positive cells is shown in the right panel. (**D**) Protein expression of p21, p16, and K9M-H3 determined by Western blotting (left). Quantitation is shown on the right. * *p* < 0.05, ** *p* < 0.01 vs. AAPH treated group.

Based on the activities of BLF reported, we hypothesized that BLF may inhibit AAPH-induced senescence. Both replicative and premature senescent cells show significant proliferation inhibition. As shown in Figure 1A, BLF at 5–80 μg/mL significantly attenuated the proliferation inhibitory effect of AAPH in HaCaT cells. However, the inhibitory effect on HaCaT cell proliferation was enhanced when the concentration was 160 μg/mL. Cell cycle arrest is a key feature of cellular senescence; cell cycle regulators such as p16, p21, and p53 are commonly used to detect senescent cells. Lamin B1 is closely related to senescence. As shown in Figure 1B, 10–40 μg/mL BLF could significantly reduce the gene expression levels of p53, p21, and p16 and increase the gene expression level of Lamin B1. Senescence-associated heterochromatin foci (SAHF) can be visualized by staining with DAPI and antibody against K9 trimethylated histone 3 (K9M-H3). Further results showed that BLF could significantly reduce the SAHF-positive cell rate and the protein expression levels of p21, p16, and K9M-H3 induced by AAPH (Figure 1C,D). These findings suggested that BLF could inhibit oxidative stress-induced senescence.

### 3.2. The Anti-Senescence Effect of BLF Is Related to the Antioxidant and Anti-Inflammatory Abilities

**Figure 2:**
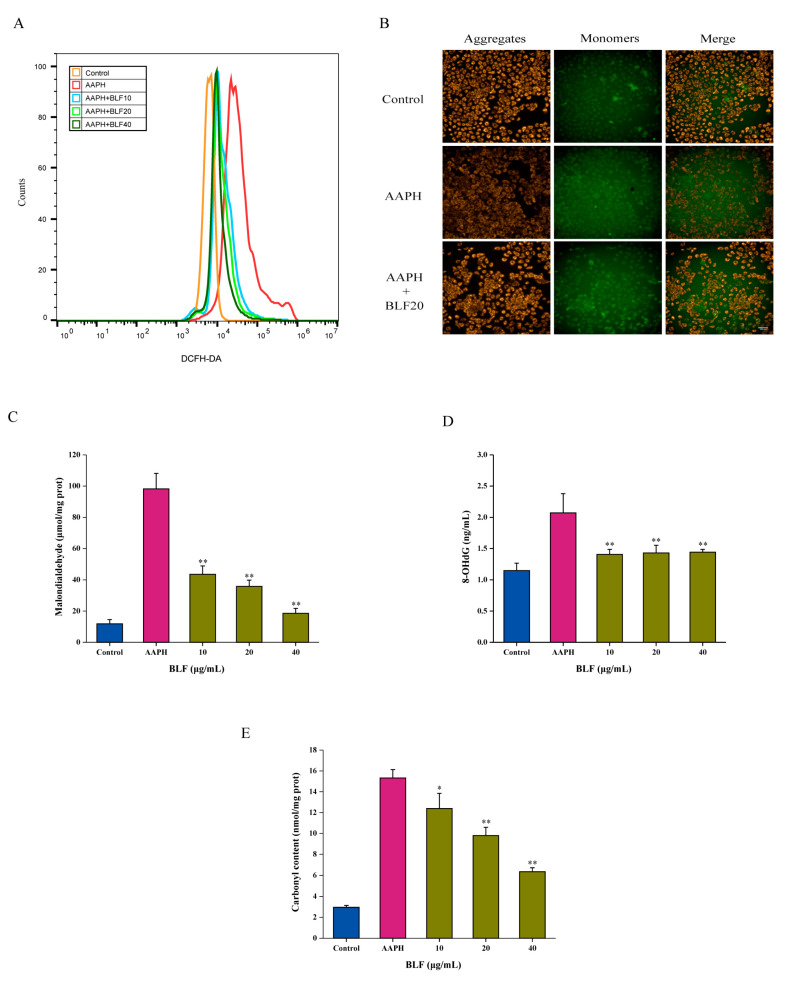
BLF inhibited AAPH-induced oxidative stress. (**A**) ROS level measured by DCFH-DA staining and detected by flow cytometry. (**B**) Representative images of mitochondrial membrane potential. Scale bar = 100 μm. (**C**) Malondialdehyde detected using lipid oxidation (MDA) assay kit. (**D**) 8-hydroxy-2′-deoxyguanosine detected using human 8-OHdG ELISA kit. (**E**) Protein carbonylation detected using protein carbonyl content detection kit. * *p* < 0.05, ** *p* < 0.01 vs. AAPH-treated group.

**Figure 3:**
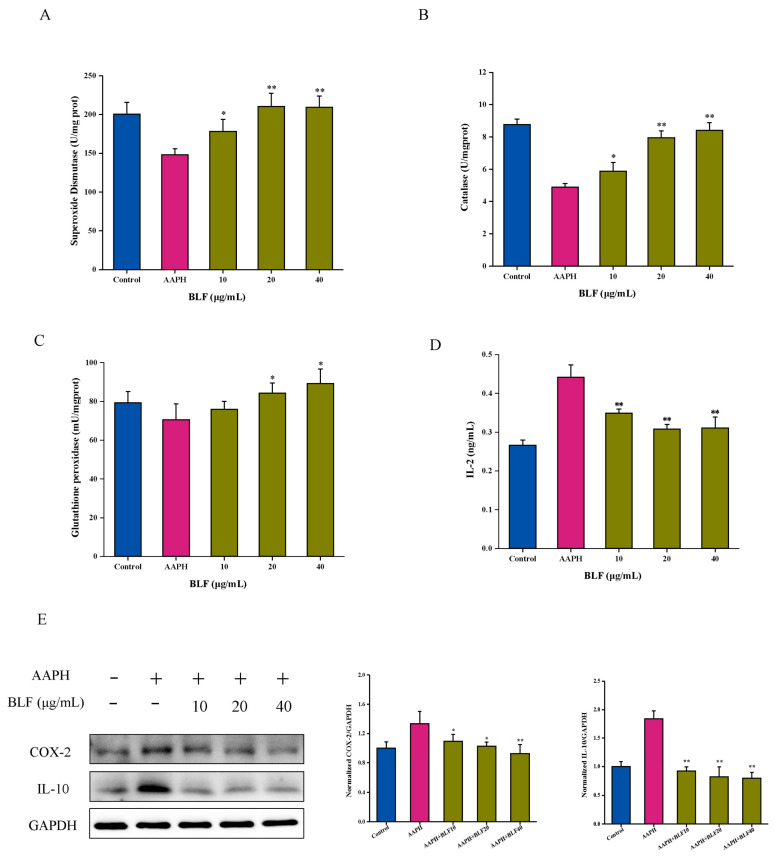
BLF increased the activity of antioxidant enzymes and inhibited the expression of inflammatory factors. (**A**) Superoxide dismutase determined by total SOD assay kit with WST-8 and detected by microplate reader. (**B**) Catalase determined by catalase assay kit. (**C**) Glutathione peroxidase determined by cellular glutathione peroxidase assay kit with NADPH. (**D**) IL-2 detected using human IL-2 ELISA kit. (**E**) Protein expression of COX-2 and IL-10 determined by Western blotting (left). Quantitation is shown on the right. * *p* < 0.05, ** *p* < 0.01 vs. AAPH treated group.

The improvement of cognitive impairment and cardiovascular protection of BLF benefited from their antioxidant and anti-inflammatory abilities; we asked whether BLF could inhibit the effect of AAPH by reducing oxidative stress and inflammation. As shown in Figure 2A, 1 mM of AAPH increased the ROS level in HaCaT cells, which was reduced by BLF in a dose-dependent manner. It was accompanied by the recovery of mitochondrial membrane potential (Figure 2B). In addition, BLF could significantly reduce the content of MDA, 8-OHdG, and protein carbonylation induced by AAPH (Figure 2C–E). Antioxidant enzymes are essential for the removal of ROS. BLF could significantly inhibit AAPH-induced oxidative stress by increasing the activity of superoxide dismutase, glutathione peroxidase, and catalase (Figure 3A–C). Further data showed that AAPH could induce the expression of IL-2, IL-10, and COX-2 of HaCaT cells, which could be significantly suppressed by the BLF (Figure 3D,E).

### 3.3. BLF Inhibit Senescence by Regulating p38 MAPK Signaling

**Figure 4:**
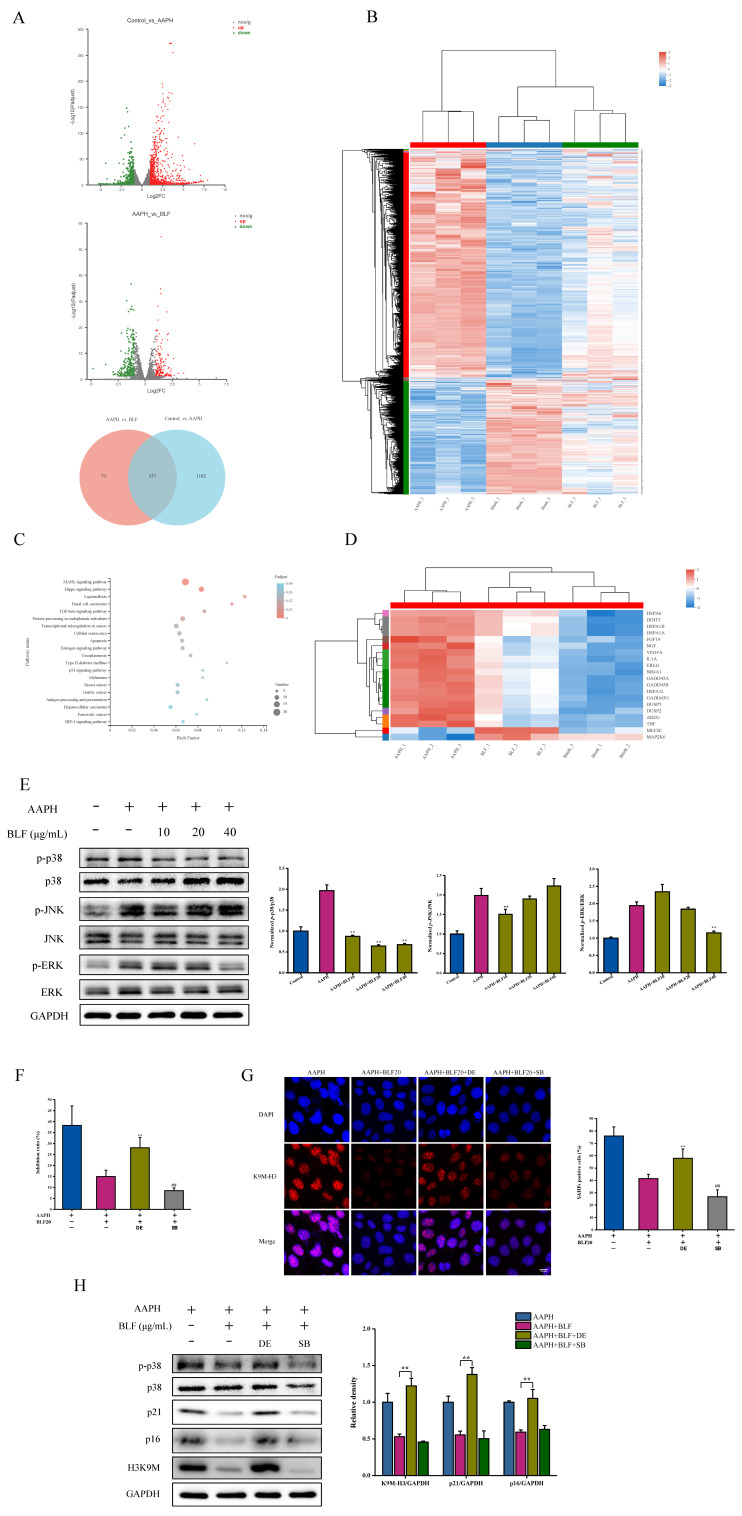
BLF inhibited AAPH-induced cellular senescence by regulating p38 MAPK signaling. (**A**) The analysis and screening of differential expressed genes (DEGs) in the control group, AAPH-treated group and AAPH and BLF co-treated group. (**B**) Cluster of DEGs in each group. (**C**) KEGG enrichment analysis of DEGs between the AAPH-treated group and AAPH and BLF co-treated group. (**D**) Normalized MAPK-related DEGs in each group. (**E**) Protein expression of p-p38, p38, p-JNK, JNK, p-ERK, and ERK determined by Western blotting (**left**). Quantitation is shown on the right. ** *p* < 0.01 vs. AAPH treated group. (**F**) HaCaT cells were pretreated with 10, 20, and 40 μg/mL BLF, 100 nM dehydrocorydaline (DE), or 10 μM SB203580 (SB) for 1 h, and then co-incubated with 1 mM AAPH for another 48 h. The proliferation inhibition of HaCaT cells determined by the MTT assay after DE and SB treatment in HaCaT cells. (**G**) Representative images of SAHF formation photographed by confocal microscope. Scale bar = 10 μm. The percentage of SAHF positive cells is shown in the right panel. (**H**) Protein expression of p21, p16, and K9M-H3 determined by Western blotting after DE and SB treatment in HaCaT cells. (**left**). Quantitation is shown on the right. ## *p* < 0.01, ** *p* < 0.01 vs. AAPH-treated group.

mRNA sequencing was performed on the following groups: control HaCaT cells, AAPH-treated HaCaT cells, and AAPH and BLF co-treated HaCaT cells. Figure 4A revealed the following: compared to the control group, AAPH induced 1519 differentially expressed genes (DEGs); and 412 DEGs existed between the AAPH group and the AAPH + BLF group. GO and KEGG enrichment analysis suggested that the MAPK pathways were most significantly enriched in the DEGs between the AAPH group and the AAPH + BLF group (Figure 4B,C and Figures S4–S6). The relative expression levels of differentially expressed MAPK genes in each group are shown in Figure 4D. Next, we further verified the sequencing results. The MAPK signaling pathway mainly includes p38, c-Jun N-terminal kinase (JNK), and the extracellular regulatory protein kinase (ERK) pathway. The results of Western blotting showed that AAPH treatment increased the phosphorylation level of p38, JNK, and ERK, and BLF could significantly reduce the phosphorylation level of p38 in a dose-dependent manner without affecting the phosphorylation level of ERK and JNK (Figure 4E). Subsequently, we respectively added the p38 specific inhibitor SB and the activator DE to co-treat HaCaT cells with BLF for 48 h. The results showed that DE could restore the phosphorylation of p38 and counteract the anti-senescence effect of BLF, which was manifested by the increase in cell proliferation inhibition rate, the number of SAHF-positive cells, and the protein levels of p21, p16, and K9M-H3. In addition, SB could enhance the anti-senescence effect of BLF (Figure 4F–H).

### 3.4. Autophagy Participates in the Anti-Senescence Effect of BLF and Is Regulated by p38 MAPK

**Figure 5:**
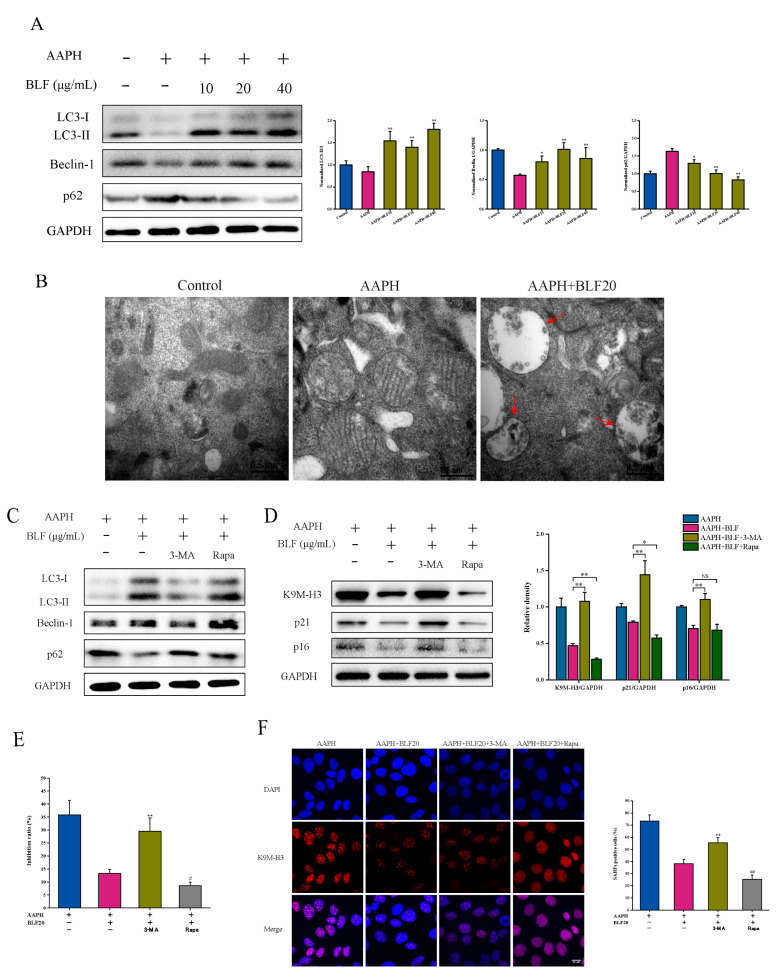
Autophagy participated in the anti-senescence effect of BLF. (**A**) Protein expression of LC3-I/II, Beclin-1, and p62 determined by Western blotting (**left**). Quantitation is shown on the right. * *p* < 0.05, ** *p* < 0.01 vs. AAPH+BLF group. (**B**) Autophagosomes detected by TEM. Representative images are shown. Scale bar = 0.5 μm. (**C**) HaCaT cells were pretreated with 20 μg/mL BLF, 100 nM Rapa, or 5 mM 3-MA for 1 h and then co-incubated with 1 mM AAPH for another 48 h. Protein expression of LC3-I/II, Beclin-1, and p62 determined by Western blotting after 3-MA and Rapa treatment in HaCaT cells. (**D**) Protein expression of p21, p16, and K9M-H3 determined by Western blotting after 3-MA and Rapa treatment in HaCaT cells (**left**). Quantitation is shown on the right. * *p* < 0.05, ** *p* < 0.01. NS, no significant difference. (**E**) The proliferation inhibition of HaCaT cells determined by the MTT assay after 3-MA and Rapa treatment in HaCaT cells, # *p* < 0.05, ## *p* < 0.01. (**F**) Representative images of SAHF formation photographed by confocal microscope after 3-MA and Rapa treatment in HaCaT cells. Scale bar = 10 μm. The percentage of SAHF positive cells is shown in the right.

Autophagy is closely related to aging. Isoorientin, one of the BLF, has been proved to regulate autophagy. We further analyzed whether BLF could also participate in the regulation of senescence of HaCaT cells through autophagy. The expression of LC3II is proportional to the number of autophagic vacuoles. Beclin-1 and p62 are key proteins in the process of autophagy. Compared with the control group, 1 mM AAPH reduced the protein expression level of Beclin-1 and increased the protein expression level of p62. Meanwhile, 10–40 μg/mL BLF could significantly reduce the level of p62 while increasing the level of Beclin-1 and LCI/II conversion (Figure 5A). In addition, we detected the formation of autophagosomes by TEM. The control group showed normal mitochondria, while the mitochondria in the AAPH treatment group were swollen, and autophagosomes were found after BLF treatment (Figure 5B). These data suggested that BLF could inhibit oxidative stress-induced senescence by activating autophagy. The addition of autophagy inhibitor 3-MA and 20 μg/mL BLF co-incubation inhibited the anti-senescence effect of BLF while inhibiting autophagy, which was manifested by restoring the formation of SAHF and increasing the protein levels of p21, p16, and K9M-H3. Furthermore, co-treatment with the autophagy inducer Rapa could further enhance the effect of BLF (Figure 5C–F).

**Figure 6:**
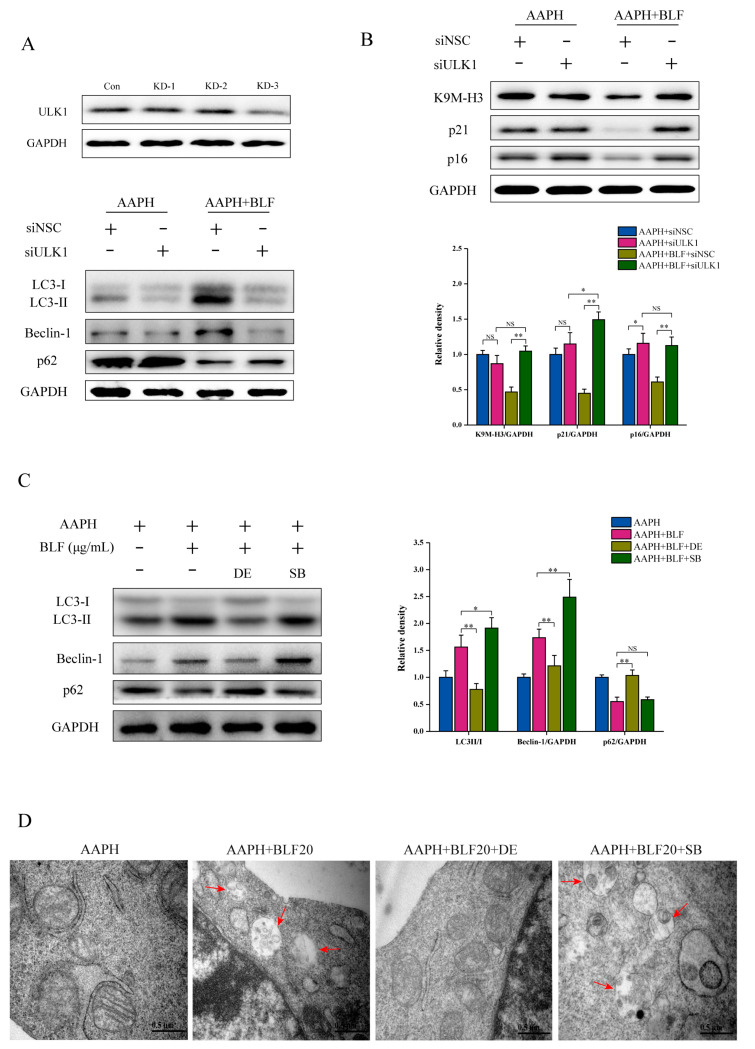
Autophagy participated in the anti-senescence effect of BLF and was regulated by p38 MAPK. (**A**) Silencing efficiency of siRNA targeting ULK1 evaluated by Western blotting. (**B**) Protein expression of p21, p16, and K9M-H3 determined by Western blotting in HaCaT cells transfected with control siRNA or siRNA targeting ULK1. Quantitation is shown below. (**C**) Protein expression of C3-I/II, Beclin-1, and p62 determined by Western blotting after DE and SB treatment in HaCaT cells. (**left**). Quantitation is shown on the right. * *p* < 0.05, ** *p* < 0.01. NS, no significant difference. (**D**) Autophagosomes detected by TEM after DE and SB treatment in HaCaT cells. Representative images are shown. Scale bar = 0.5 μm.

The ULK1 complex plays an important role in autophagy initiation. In order to further confirm the role of autophagy in the anti-senescence effect of BLF, we used siRNA to knock down the expression of ULK1. Figure 6A,B show that the inhibition of ULK1 eliminated the regulatory effect of BLF. These results proved that autophagy participated in the anti-senescence effect of BLF. The MAPK signaling pathway may be a potential junction of oxidative stress-induced skin aging and autophagy. We further analyzed whether BLF-activated autophagy was regulated by p38 MAPK. The results showed that the p38 activator DE could significantly inhibit the induction of autophagy by BLF, which was manifested by increasing the expression level of p62 and reducing the level of Beclin-1 and LCI/II conversion. Co-treatment with the p38 inhibitor SB could further enhance the induction of autophagy (Figure 6C,D).

### 3.5. BLF Delays UVB-Induced Dorsal Skin Aging in Mice

**Figure 7:**
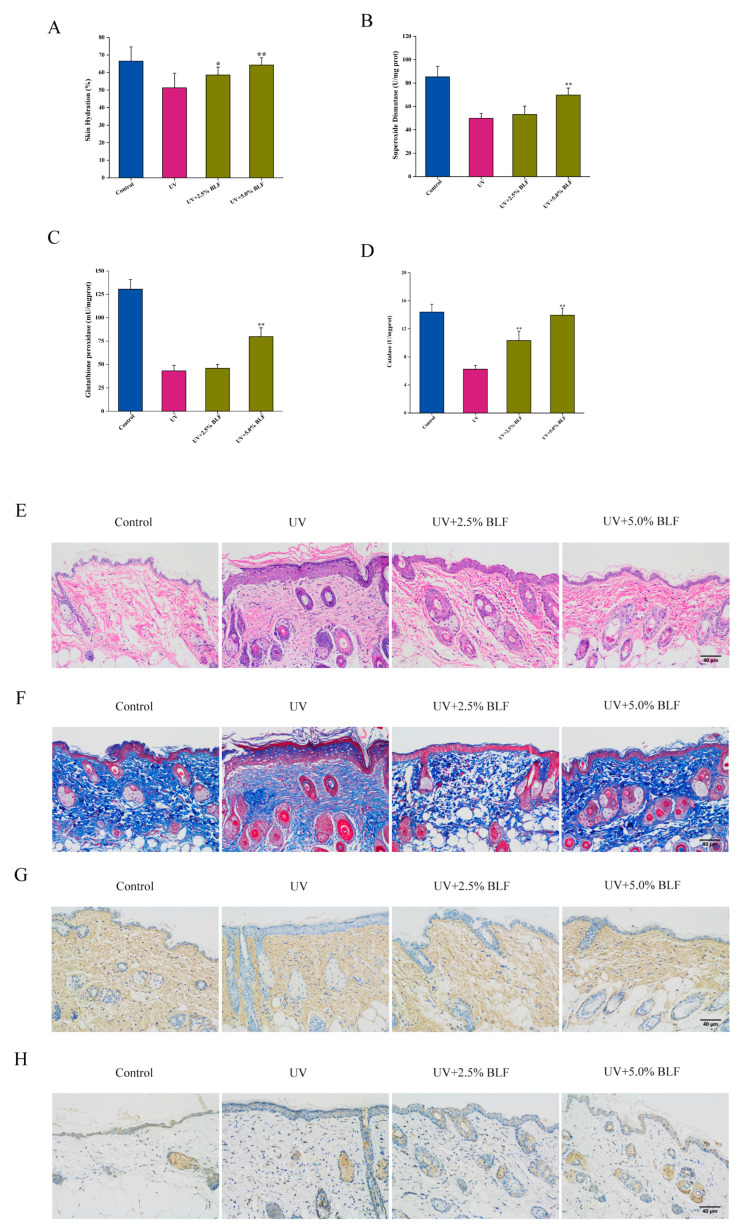
BLF delayed UVB-induced dorsal skin aging in mice. (**A**) Skin hydration analysis. (**B**) Superoxide dismutase determined by a total SOD assay kit with WST-8 and detected by a microplate reader. (**C**) Glutathione peroxidase determined by a cellular glutathione peroxidase assay kit with NADPH. (**D**) Catalase determined by a catalase assay kit. * *p* < 0.05, ** *p* < 0.01 vs. UV group. (**E**) Epidermal thickness was observed by H&E staining. (**F**) Collagen fibers were stained by Masson staining. (**G**) Type I collagen protein level in skin tissue was detected by immunohistochemistry. (**H**) MMP-3 protein level in skin tissue was detected by immunohistochemistry. Representative images are shown (Scale bar = 40 μm).

**Figure 8:**
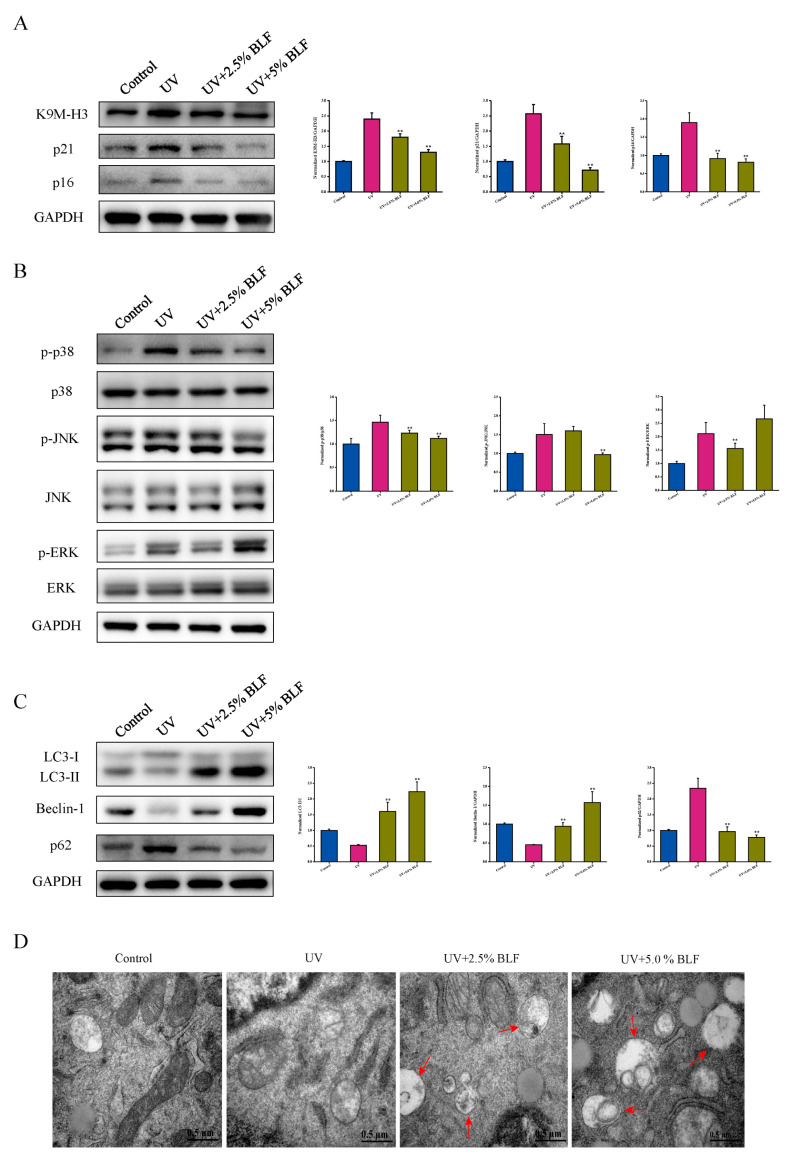
BLF delayed UVB-induced dorsal skin aging in mice. (**A**) Protein expression of K9M-H3, p21, and p16 determined by Western blotting. (**B**) Protein expression of p-p38, p38, p-JNK, JNK, p-ERK, and ERK determined by Western blotting. (**C**) Protein expression of LC3-I/II, Beclin-1, and p62 determined by Western blotting. Quantitation is shown on the right. (**D**) Autophagosomes detected by TEM in skin tissue. Representative images are shown. Scale bar = 0.5 μm. ** *p* < 0.01 vs. UV group.

Although the anti-senescence effect of BLF has been proven in the oxidative stress-induced cellular senescence model, it is necessary to further verify whether BLF can alleviate skin aging in vivo. Ultraviolet radiation is an independent cause of accelerated skin aging. Our results showed that BLF treatment significantly restored skin hydration and increased the activity of superoxide dismutase, glutathione peroxidase, and catalase (Figure 7A–D). The results of histopathological analysis showed that UVB irradiation increased epidermal thickness and decreased the content of collagen fiber and type I collagen in the dorsal skin of mice. In addition, UVB irradiation also increased the expression of MMP-3, which is a major participant in collagen breakdown. In contrast, BLF treatment reduced epidermal thickness and increased collagen fiber content while inhibiting MMP-3 expression. However, BLF did not improve the expression of type I collagen (Figure 7E–H). Notably, BLF inhibited the increase in p16, p21, and K9M-H3 protein levels in skin tissues caused by UVB irradiation (Figure 8A). These data indicated that BLF could protect the dorsal skin of mice from UVB-induced aging, and BLF could restore p38 phosphorylation induced by UVB radiation (Figure 8B). Furthermore, we found that BLF reduced the protein expression level of p62, increased the protein expression level of Beclin-1, and promoted LC3 I/II conversion in skin tissue (Figure 8C). Lastly, TEM imaging of skin tissue showed that autophagosomes were abundant after BLF treatment (Figure 8D). These data provided in vivo evidence for BLF to delay skin aging caused by UVB irradiation.

## 4. Discussion

Previous studies have shown that BLF have a variety health and medicinal values. In the present study, the inhibitory effect of BLF as a potential therapeutic agent on skin senescent cells was first evaluated. Senescent cells can break away from the cell cycle and lose the ability to proliferate in response to growth factors or mitogens [26]. Therefore, cell cycle regulators such as p53, p21, and p16 are important biomarkers of cellular senescence. The expression of p21 and p53 increased significantly in an AAPH-induced senescence model of transformed skin cells and normal human epidermal keratinocytes [10]. The same phenomenon appeared in the UVB-induced senescence model of human skin fibroblasts [27]. In addition, cellular senescence is often accompanied by changes in chromatin structure and the appearance of punctate heterochromatin structure, which is known as SAHF [1,28]. Lamin B1 is involved in the formation of SAHF and is also one of the biomarkers of senescence and aging [29]. The detection of the above biomarkers of senescence confirmed the anti-senescence effect of BLF.

According to the free radical aging theory, excessive ROS induce oxidative stress to directly damage skin cells or mediate inflammatory response and collagen synthesis and decomposition by regulating MAPK and NF-κB signaling pathways in the process of skin aging. Cellular senescence can promote the transformation of skin benign papillomas to carcinomas by enhancing p38 MAPK and ERK MAPK signaling [30]. The hyperoside-enriched fraction prepared from Houttuynia cordata Thunb can regulate the MAPK signaling by attenuating the activation of JNK/ERK/c-Jun in human skin fibroblasts to inhibit skin aging induced by UVB irradiation [31]. The detection of oxidative damage indicators and inflammatory factors proved that the anti-senescence effect of BLF was at least partly due to their antioxidant and anti-inflammatory properties. In addition, an important aspect of our study was to prove that the inhibition of p38 MAPK was related to the therapeutic intervention of BLF.

Autophagy is an important way for the removal of damaged substances in cells, which can maintain the balance of cell metabolism and participate in various physiological and pathological processes related to aging. Although there are different opinions, most studies support the conclusion that the activation of autophagy can delay the occurrence of aging, and the crosstalk between cellular senescence and autophagy has been widely reported. The overexpression of Atg5-induced autophagy can inhibit the senescence of mouse embryonic fibroblasts [32]. p62 is a ubiquitin-binding protein, which is negatively related to autophagy to a certain extent [33]. Beclin-1 is a component of the class III PI3 kinase complex and is responsible for various stimuli such as nutrient deficiency and starvation to initiate autophagy [34]. The soluble form of LC3-I is converted to the fat-soluble form of LC3-II and is recruited to the bilayer membrane of the autophagy precursor to activate autophagy, which is one of the biomarkers of autophagy [35]. The ULK1 complex plays an important role in autophagosome maturation as well as initiation [36]. In our results, we could clearly observe the regulation effect of BLF on the protein expression of p62, Beclin-1, and LC3II and the formation of autophagosomes, which proved that autophagy was involved in the anti-senescence effect of BLF. Moreover, the anti-senescence effect of BLF was weakened after the inhibition of autophagy with 3-MA and siULK1. In addition, the topical application of BLF on the dorsal skin of UVB-irradiated mice also proved the activation of autophagy.

MAPK signaling may be a potential crosstalk pathway between senescence and autophagy [1]. p38 is closely related to the regulation of ULK1 [37]. In addition, the inhibition of p38 MAPK signaling in senescent CD8+ T cells can induce autophagy in an mTOR-independent manner [38]. Our results indicated that the activation or inhibition of p38 MAPK signaling was sufficient to change autophagy, suggesting that p38 MAPK should be the main upstream signaling in BLF-induced autophagy. However, the mechanism of whether MAPK directly regulates autophagy or how is unclear. Future work needs to focus on the molecular cascade process of MAPK regulating autophagy. The selective degradation of damaged mitochondria through autophagy is also known as mitophagy, which is closely related to ROS and mitochondrial membrane potential [39]. Our data clearly showed that BLF significantly eliminated AAPH-induced mitochondrial dysfunction by improving mitochondrial membrane potential and reducing intracellular ROS levels. However, whether BLF interferes with senescence through mitophagy has not been verified. In addition, whether the induction of autophagy can directly reduce the level of oxidative stress needs further research.

In this study, we demonstrated that BLF can inhibit oxidative stress-induced cellular senescence by regulating the p38 MAPK and autophagy pathway. In addition, we explored and verified the anti-aging effect of BLF in vivo through the skin aging model induced by ultraviolet radiation in mice. Therefore, our study shows that BLF can serve as a potential therapeutic agent for skin aging, and the effectiveness of BLF requires further clinical trials to verify.

## Data Availability

The data presented in this study are available on request from the corresponding author.

## Supplementary Materials

The following supporting information can be downloaded at: https://www.mdpi.com/article/10.3390/nu14040793/s1, Figure S1: The chemical structure of orientin, isoorientin, vitexin, and isovitexin; Figure S2: Liquid chromatogram of bamboo leaf flavonoids prepared in our laboratory; Figure S3: Correlation and PCA analysis among samples; Figure S4: GO analysis between control and AAPH groups; Figure S5: GO analysis between AAPH and AAPH + BLF groups; Figure S6: KEGG analysis between control and AAPH groups; Table S1: Formulation of emulsion.

## References

[1] Gu Y., Han J., Jiang C., Zhang Y. (2020). Biomarkers, oxidative stress and autophagy in skin aging. Ageing Res. Rev..

[2] Morita A., Torii K., Maeda A., Yamaguchi Y. (2009). Molecular basis of tobacco smoke-induced premature skin aging. J. Investig. Dermatol. Symp. Proc..

[3] Panich U., Sittithumcharee G., Rathviboon N., Jirawatnotai S. (2016). Ultraviolet Radiation-Induced Skin Aging: The Role of DNA Damage and Oxidative Stress in Epidermal Stem Cell Damage Mediated Skin Aging. Stem Cells Int..

[4] Pomatto L.C.D., Davies K.J.A. (2018). Adaptive homeostasis and the free radical theory of ageing. Free Radic. Biol. Med..

[5] Mecocci P., Boccardi V., Cecchetti R., Bastiani P., Scamosci M., Ruggiero C., Baroni M. (2018). A Long Journey into Aging, Brain Aging, and Alzheimer’s Disease Following the Oxidative Stress Tracks. J. Alzheimers Dis..

[6] Fedorova M., Bollineni R.C., Hoffmann R. (2014). Protein carbonylation as a major hallmark of oxidative damage: Update of analytical strategies. Mass Spectrom. Rev..

[7] Chen T., Hou H., Fan Y., Wang S., Chen Q., Si L., Li B. (2016). Protective effect of gelatin peptides from pacific cod skin against photoaging by inhibiting the expression of MMPs via MAPK signaling pathway. J. Photochem. Photobiol. B Biol..

[8] Wang Y., Wang L., Wen X., Hao D., Zhang N., He G., Jiang X. (2019). NF-κB signaling in skin aging. Mech. Ageing Dev..

[9] Wu G., Li S., Qu G., Hua J., Zong J., Li X., Xu F. (2021). Genistein alleviates H_2_O_2_-induced senescence of human umbilical vein endothelial cells via regulating the TXNIP/NLRP3 axis. Pharm. Biol..

[10] Li Y.F., Ouyang S.H., Tu L.F., Wang X., Yuan W.L., Wang G.E., Wu Y.P., Duan W.J., Yu H.M., Fang Z.Z. (2018). Caffeine protects skin from oxidative stress-induced senescence through the activation of autophagy. Theranostics.

[11] Mizushima N. (2018). A brief history of autophagy from cell biology to physiology and disease. Nat. Cell Biol..

[12] Wong S.Q., Kumar A.V., Mills J., Lapierre L.R. (2020). Autophagy in aging and longevity. Hum. Genet..

[13] Song X., Narzt M.S., Nagelreiter I.M., Hohensinner P., Terlecki-Zaniewicz L., Tschachler E., Grillari J., Gruber F. (2017). Autophagy deficient keratinocytes display increased DNA damage, senescence and aberrant lipid composition after oxidative stress in vitro and in vivo. Redox Biol..

[14] Kang H.T., Lee K.B., Kim S.Y., Choi H.R., Park S.C. (2011). Autophagy impairment induces premature senescence in primary human fibroblasts. PLoS ONE.

[15] Wen W., Chen J., Ding L., Luo X., Zheng X., Dai Q., Gu Q., Liu C., Liang M., Guo X. (2018). Astragaloside exerts anti-photoaging effects in UVB-induced premature senescence of rat dermal fibroblasts through enhanced autophagy. Arch. Biochem. Biophys..

[16] Wang J., Li J., Cao N., Li Z., Han J., Li L. (2018). Resveratrol, an activator of SIRT1, induces protective autophagy in non-small-cell lung cancer via inhibiting Akt/mTOR and activating p38-MAPK. OncoTargets Ther..

[17] Hou L.-J., Xie M.-Y., Ye W.-C., Zhao G.-J. (2021). Doxycycline ameliorates autophagy by inhibiting p38 MAPK in cardiac myocytes. Int. J. Cardiol..

[18] Yang J.-P., He H., Lu Y.-H. (2014). Four flavonoid compounds from Phyllostachys edulis leaf extract retard the digestion of starch and its working mechanisms. J. Agric. Food Chem..

[19] Nirmala C., Bisht M.S., Bajwa H.K., Santosh O. (2018). Bamboo: A rich source of natural antioxidants and its applications in the food and pharmaceutical industry. Trends Food Sci. Technol..

[20] Gong J., Xia D., Huang J., Ge Q., Mao J., Liu S., Zhang Y. (2015). Functional components of bamboo shavings and bamboo leaf extracts and their antioxidant activities in vitro. J. Med. Food.

[21] Yoshizaki N., Fujii T., Masaki H., Okubo T., Shimada K., Hashizume R. (2014). Orange peel extract, containing high levels of polymethoxyflavonoid, suppressed UVB-induced COX-2 expression and PGE2 production in HaCaT cells through PPAR-γ activation. Exp. Dermatol..

[22] Fan J., Zhuang Y., Li B. (2013). Effects of collagen and collagen hydrolysate from jellyfish umbrella on histological and immunity changes of mice photoaging. Nutrients.

[23] Song H., Zhang S., Zhang L., Li B. (2017). Effect of Orally Administered Collagen Peptides from Bovine Bone on Skin Aging in Chronologically Aged Mice. Nutrients.

[24] Kuo Y.-H., Chen C.-W., Chu Y., Lin P., Chiang H.-M. (2015). In Vitro and In Vivo Studies on Protective Action of N-Phenethyl Caffeamide against Photodamage of Skin. PLoS ONE.

[25] Kuo Y.-H., Lin T.-Y., You Y.-J., Wen K.-C., Sung P.-J., Chiang H.-M. (2016). Antiinflammatory and Antiphotodamaging Effects of Ergostatrien-3β-ol, Isolated from Antrodia camphorata, on Hairless Mouse Skin. Molecules.

[26] Calcinotto A., Kohli J., Zagato E., Pellegrini L., Demaria M., Alimonti A. (2019). Cellular Senescence: Aging, Cancer, and Injury. Physiol. Rev..

[27] Chen W., Kang J., Xia J., Li Y., Yang B., Chen B., Sun W., Song X., Xiang W., Wang X. (2008). p53-related apoptosis resistance and tumor suppression activity in UVB-induced premature senescent human skin fibroblasts. Int. J. Mol. Med..

[28] Sun Y., Zheng Y., Wang C., Liu Y. (2018). Glutathione depletion induces ferroptosis, autophagy, and premature cell senescence in retinal pigment epithelial cells. Cell Death Dis..

[29] Wang A.S., Ong P.F., Chojnowski A., Clavel C., Dreesen O. (2017). Loss of lamin B1 is a biomarker to quantify cellular senescence in photoaged skin. Sci. Rep..

[30] Alimirah F., Pulido T., Valdovinos A., Alptekin S., Chang E., Jones E., Diaz D.A., Flores J., Velarde M.C., Demaria M. (2020). Cellular Senescence Promotes Skin Carcinogenesis through p38MAPK and p44/42MAPK Signaling. Cancer Res..

[31] Mapoung S., Umsumarng S., Semmarath W., Arjsri P., Srisawad K., Thippraphan P., Yodkeeree S., Dejkriengkraikul P. (2021). Photoprotective Effects of a Hyperoside-Enriched Fraction Prepared from Houttuynia cordata Thunb. on Ultraviolet B-Induced Skin Aging in Human Fibroblasts through the MAPK Signaling Pathway. Plants.

[32] Wang Y., Wang X., Lapi E., Sullivan A., Jia W., He Y.-W., Ratnayaka I., Zhong S., Goldin R., Goemans C. (2012). Autophagic activity dictates the cellular response to oncogenic RAS. Proc. Natl. Acad. Sci. USA.

[33] Deng Z., Lim J., Wang Q., Purtell K., Wu S., Palomo G.M., Tan H., Manfredi G., Zhao Y., Peng J. (2020). ALS-FTLD-linked mutations of SQSTM1/p62 disrupt selective autophagy and NFE2L2/NRF2 anti-oxidative stress pathway. Autophagy.

[34] Xu H.-D., Qin Z.-H. (2019). Beclin 1, Bcl-2 and Autophagy. Adv. Exp. Med. Biol..

[35] Kharaziha P., Panaretakis T. (2017). Dynamics of Atg5-Atg12-Atg16L1 Aggregation and Deaggregation. Methods Enzymol..

[36] Zachari M., Ganley I.G. (2017). The mammalian ULK1 complex and autophagy initiation. Essays Biochem..

[37] Slobodnyuk K., Radic N., Ivanova S., Llado A., Trempolec N., Zorzano A., Nebreda A.R. (2019). Autophagy-induced senescence is regulated by p38α signaling. Cell Death Dis..

[38] Henson S.M., Lanna A., Riddell N.E., Franzese O., Macaulay R., Griffiths S.J., Puleston D.J., Watson A.S., Simon A.K., Tooze S.A. (2014). p38 signaling inhibits mTORC1-independent autophagy in senescent human CD8^+^ T cells. J. Clin. Investig..

[39] Baechler B.L., Bloemberg D., Quadrilatero J. (2019). Mitophagy regulates mitochondrial network signaling, oxidative stress, and apoptosis during myoblast differentiation. Autophagy.

