# Supplementary material for: Bamboo Leaf Flavonoids Suppress Oxidative Stress-Induced Senescence of HaCaT Cells and UVB-Induced Photoaging of Mice through p38 MAPK and Autophagy Signaling
Source: Nutrients. 2022 Feb 14;14(4):793. doi: 10.3390/nu14040793 (PMC8876272; doi:10.3390/nu14040793)
Supplement: Supplementary file 1 [file nutrients-14-00793-s001.zip › nutrients-1564496-supplementary.pdf]

Bamboo leaf flavonoids suppress oxidative stress-induced senescence of HaCaT cells and UVB-induced photoaging of mice through p38 MAPK and autophagy signaling

Table S1 Formulation of emulsion. Mix the components of phase A and phase B uniformly. After heating to 80℃, slowly pour phase A into phase B. After homogenizing (2000 rpm/min, 5 min), stir and cool down to 40℃, then add phase C and D in sequence, stir evenly.

Phase	Component	Mass fraction (%)
A	Isopropyl myristate	3.00
	Polydimethylsiloxane	3.00
	Liquid paraffin	1.00
	Glyceryl stearatese	1.00
	Cetearyl alcohol	1.50
	Sucrose stearate ester	2.00
B	Glycerin	4.00
	Butanediol	3.00
	Xanthan gum	0.10
	EDTA-2Na	0.03
	Pure water	to 100
C	Methyl isothiazolinone	0.10
D	BLF	2.5, 5.0

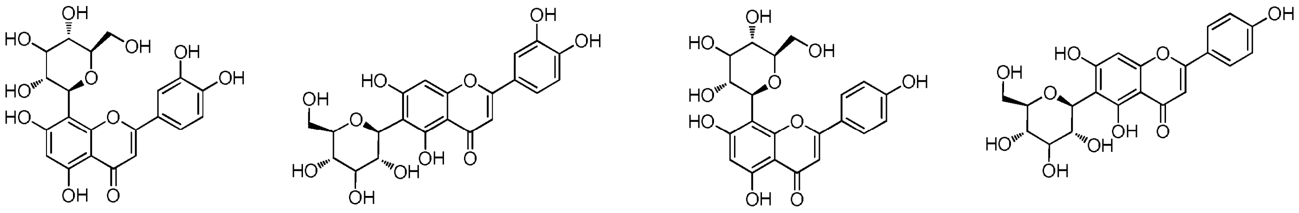

Orientin Isoorientin Vitexin Isovitexin

Figure S1 The chemical structure of orientin, isoorientin, vitexin and isovitexin.

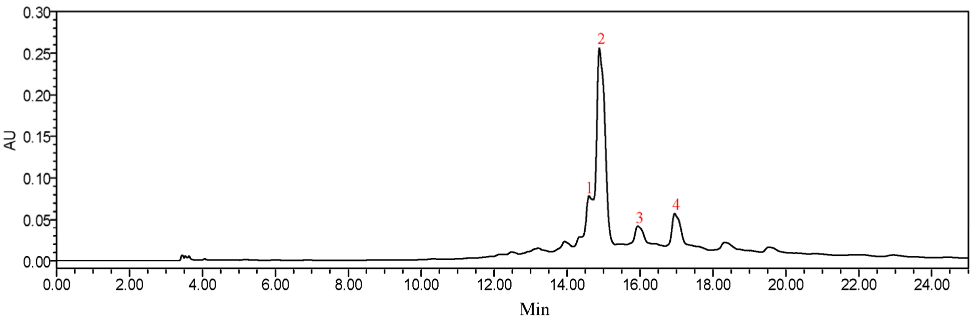

Peak	Flavonoid compound	Retention time	Area	Height	% Area
1	isoorientin	14.680	1028577	63027	10.64
2	orientin	14.965	3956320	214205	40.92
3	isovitexin	16.019	1511659	34918	15.63
4	vitexin	17.033	1302318	45940	13.47

Figure S2 Liquid chromatogram of bamboo leaf flavonoids prepared in our laboratory.

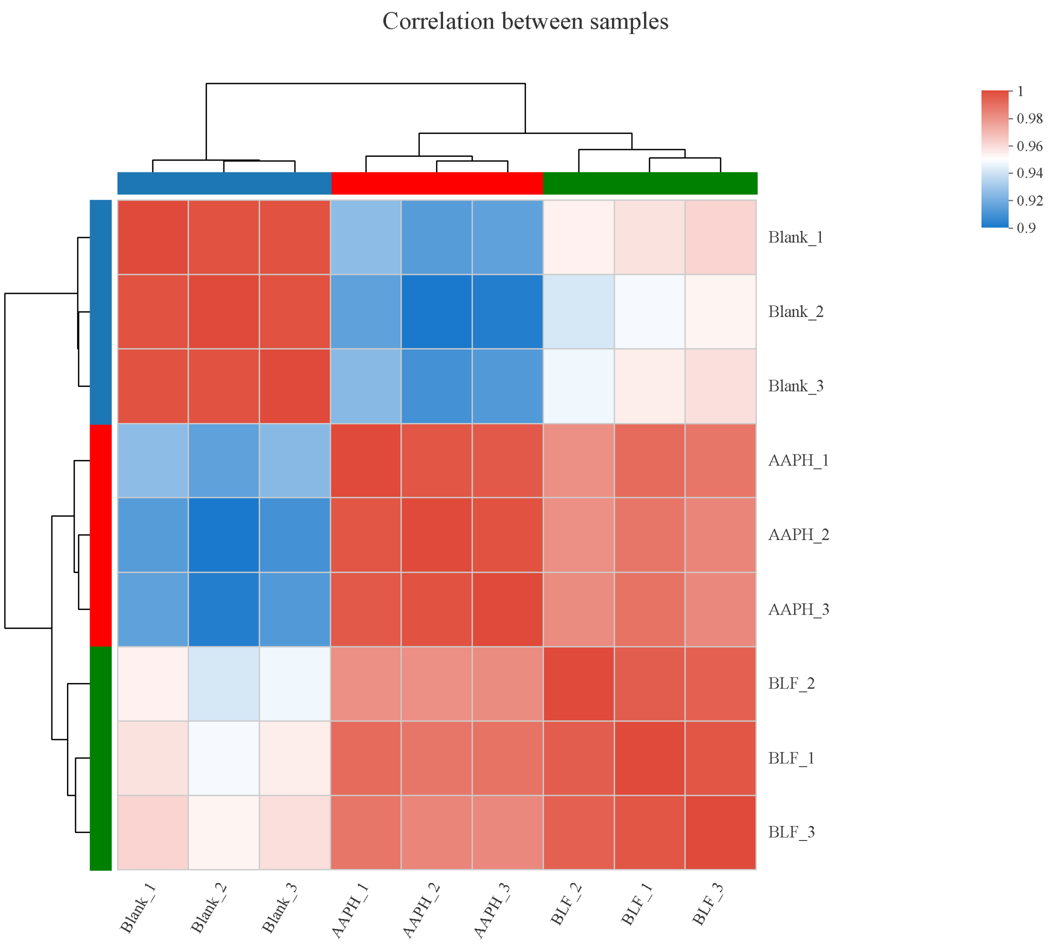

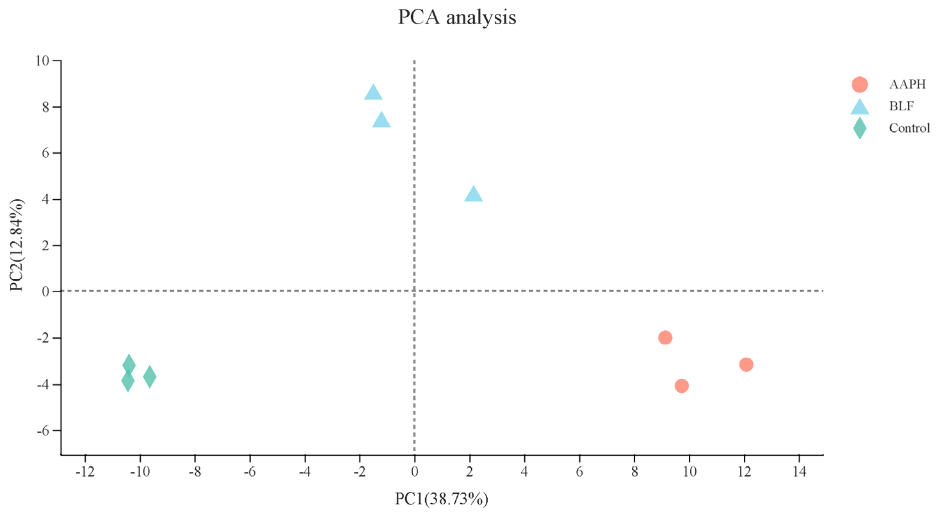

Figure S3 Correlation and PCA analysis among samples.

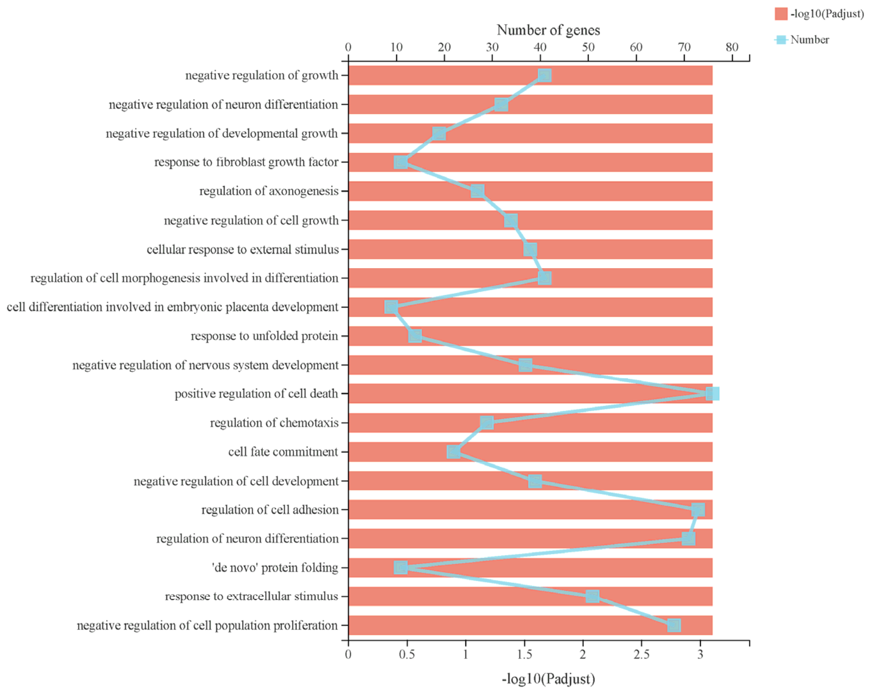

Figure S4 GO analysis between control and AAPH groups.

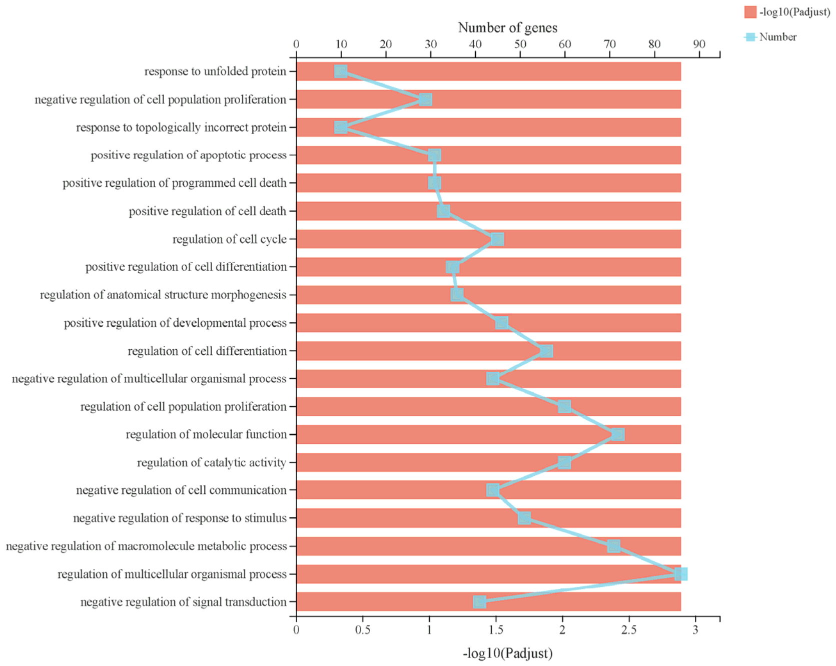

Figure S5 GO analysis between AAPH and AAPH + BLF groups.

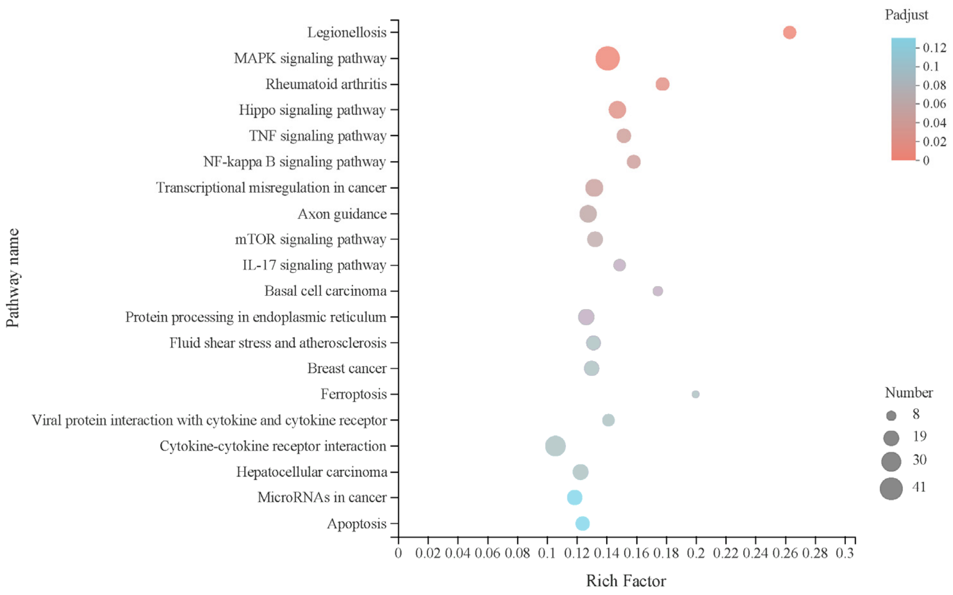

Figure S6 KEGG analysis between control and AAPH groups.
